# TMJ Dysfunctions Systemic Implications and Postural Assessments: A Review of Recent Literature

**DOI:** 10.3390/jfmk4030058

**Published:** 2019-08-19

**Authors:** Sergio Sambataro, Gabriele Cervino, Salvatore Bocchieri, Rosario La Bruna, Marco Cicciù

**Affiliations:** 1 Department of Biomedical and Dental Sciences and Morphological and Functional Imaging, Messina University, 98100 Messina, Italy; 2 Private Practice, 89100 Reggio Calabria, Italy

**Keywords:** TMJ, spinal cord, cervical, vertebra, dentistry, malocclusion, orthodontic, gnatologic, physiotherapy, orthopedic

## Abstract

Cases of correlations between posture and the temporomandibular joint have long been reported in the literature. In particular, occlusal anomalies, and therefore malocclusion, could have negative implications for the spine. The objective of this study was to review the literature and bring to light any correlations between temporomandibular joints (TMJ) and posturology. The literature search was conducted in the PubMed and Embase scientific search engines with the aim of obtaining the most possible results in the initial search, the number of results initially obtained was 263. Subsequently, the inclusion and exclusion criteria were reduced first to 83 and subsequently to manual analysis of the articles, those included remained only 11. The results show a correlation between anomalies of the TMJ and dysfunctions of the vertebral column. Not all the articles considered are in agreement with each other regarding epidemiological data, but surely this study can represent an important starting point for a much more careful evaluation of the dental patient and at the same time for the request for counseling by a dentist in case of postural abnormalities.

## 1. Introduction

The purpose of this scientific study is to highlight any clinical correlations between malocclusions, dysfunctions of the temporomandibular joint and postural abnormalities. In literature in recent years there has been much talk about posturology, often linked to figures such as osteopathy and physiotherapy. The goal is to bring to light clinical data from other studies and review them so as to make a point of the situation and have reliable information regarding these correlations. Moreover, it would be interesting to highlight if they are linked by a cause–effect relationship, and therefore intervening on the posture, on the back in any stretch, dento-occlusal consequences occur. Back pain is one of the most common pathologies of our times. This pathology contains multiple pathologies and multiple causes that can lead to the appearance of back pain symptoms. Back pain can involve different parts of the spine such as the intervertebral discs (placed between one vertebra and another), the vertebrae, the facet joints, the muscles or the capsule that surrounds the joints that connect the vertebrae and also different areas of the column as the cervical, dorsal or lumbar tract. Chiropractors were the first to deal with this subject as they realized that some of their patients did not benefit from their therapies and most of them all had an altered dental occlusion; since then many other schools of postural osteopathy, orthopedics and classical physiatrists have dealt with this fascinating subject on several occasions.

Orthodontics is the science for the correction of malocclusion of the teeth. It is taken to mean the dealing with movement of the teeth and alteration of the alveolar process supporting the teeth. It concerns also the growth and the various anomalies of development and position of the skeletal structures usually associated with the basal bone of the jaws. It deals with the nerve, muscle and all connective tissues including the joints involved with the motion of organs concerned [1,2,3].

TMJ dysfunctions are strictly related with an alteration of the position of the condyle in the glenoid fossa. Systemic factors, psychological conditions, anxiety and tension should be recognized by the clinicians. Occlusal disharmony should be ruled out as an etiological factor before radical treatment is resorted to in all TMJ patients [4,5,6,7].

TMJ surgery was performed by maxillofacial surgeons in the 80s but a lot of side effects were developed by patients and now the indications are very few: Only ankyloses of the joint or severe displaced and dislocated extra capsular condyle fractures are eligible to be treated surgically [8,9,10,11,12,13]. 

On the other hand, oral surgery procedures, like extraction of third molars or their germectomy, in some cases, helping orthodontists in preventing interferences in occlusion and avoid patients the need to be subjected to major surgery procedures and in assuming a lot of drugs and medications [14,15,16,17,18,19,20,21,22].

The objective of this study is to study the possible correlations between the temporomandibular joint and the muscle chains. Study how muscle chains interact in occlusion and how they can modify it.

## 2. Materials and Methods

### 2.1. Application Protocol and Website Recording Data

A protocol including the investigation methods and the inclusion criteria for the current revision was submitted in the PROSPERO website, an international prospective register of systematic reviews. The parameters and the analytic structure of the present work can be visualized relating the CRD ID and code; this systematic review was submitted at the PROSPERO website platform, with PROSPERO acknowledgement of receipt number 132,529.

The data of this systematic investigation observed the Preferred Reporting Items for Systematic Review in accordance with the preferred reporting items for systematic reviews and meta-analyses (PRISMA) statement.

### 2.2. Target Questions

The questions processed the following guidelines, according to PICO:

• Are there any correlations between TMJ dysfunctions and postural assessments?

### 2.3. Search Strategy

We conducted a search in five electronic databases, including Ovid MEDLINE, PubMed and EMBASE. In addition, a manual search on the Dentistry and Pharmacological source was conducted, for relevant studies published.

Digital and searches by hand were then performed in TMJ disorders and posture attitude. In-depth research of the reference lists in the recorded manuscripts was performed in order to add significant studies and to increase the sensitivity of the revision.

### 2.4. Collection Data

Medical Subject Headings (MeSH) were applied for finding the keywords used in the present revision. The selected key words: (“TMJ disorder” and “posture”) were recorded for collecting the data. The date of the last search with these results was 31 May 2019.

### 2.5. Manuscript Selections

Two independent reviewers of two different universities (Messina and Naples) singularly analyzed the obtained papers in order to select the inclusion and exclusion criteria as follows. Reviewers correlated their evaluations and analyzed differences through comparing the manuscripts and consulting a third experienced senior independent reviewer (G.C.; University of Messina) when a consensus could not be reached. For the stage of the full-text articles revision, a complete independent dual analysis was performed. 

### 2.6. Research Classifications

The method of classification included all human prospective and retrospective clinical studies, case–control papers, case series manuscripts and literature reviews published between February 2009 and May 2019, on TMJ dysfunctions related to posture.

### 2.7. Exclusion and Inclusion Criteria

The full texts of all studies related to the main revision topics were obtained for comparing the inclusion parameters:

- Investigated relations between TMJ dysfunctions and posture of the human body.
- The following exclusion criteria included:
- Not enough information regarding the topic;
- Animal or in vitro studies;
- Articles published prior to 1 February 2009;
- No access to the title and abstract.

### 2.8. Strategy for Collecting Data

After the first literature analysis, the entire manuscript titles list was highlighted to exclude irrelevant publications, case reports and the non-English language publications. Then, research was excluded based on the data obtained from screening just the abstracts. The final selection was performed reading the full texts of the papers in order to approve each study’s eligibility, based on the inclusion and exclusion criteria.

### 2.9. Record of the Extracted and Collected Data Extraction

The results and conclusions of the selected full text papers were used for assembling the data, according to the aims and themes of the present revision.

### 2.10. Risk of Bias Assessment

The grade of bias risk was independently considered, as reported in the literature [23,24,25].

Potential causes of bias were investigated:

- Selection bias;
- Performance bias and detection bias;
- Attrition bias;
- Reporting bias;
- Examiner blinding, examiner calibration, standardized follow-up description, standardized residual graft measurement and standardized radiographic assessment.

### 2.11. Occlusion

Angle defines occlusion as being the normal relations of the occlusal inclined planes of the teeth when the jaws are closed [3].

The science of occlusion is concerned with the study of the contribution of the teeth and jaws to complete physical, mental and social well-being of the patient. Thus, occlusion is more than just the fit of the teeth or “a place where the teeth meet to eat”. It involves the entire postural apparatus as the support of the skull and jaws relates to the neck and the complete body [2].

### 2.12. Malocclusion

Malocclusion is a condition in which there is a deflection from the normal relation of teeth to other teeth in the same arch and/or to teeth in the opposing arch. There are intra-arch and inter-arch problems: Inter-arch are labio-version, linguo-version, mesio-version or disto-version, rotation or transposition, instead inter arch are sagittal, transverse or vertical issue. There are different reasons why malocclusion originates, and genetics play an important role, but could be modified by the environment, particularly pathologic alteration of function became the subject of interest. It is accepted the genetics to be by far the dominant factor in the range of 80%, with function being a strong consideration, particularly when it was great enough to over-ride the genetic potential. Conditions such as thumb sucking, tongue thrusting, premature loss of teeth from an accident or dental disease and respiratory problems, as oral breathing or the permanence of hypertrophic tonsils and adenoids, labio-lingual imbalance were considerations for functional dominations. These conditions allow us to consolidate most of the malocclusions. There are different classifications of malocclusion, but the most used all around the world is the Angle’s classification, that is based on the relationship of the mesio-buccal cusp of the maxillary first molar and the buccal groove of the mandibular first molar. 

It consists of three classes:

In Class I, the mesio-buccal cusp of the maxillary first molar occluding in line with the buccal groove of the mandibular first molar i.e., the maxillary first molar is slightly posteriorly positioned relative to the mandibular first molar.

In Class 2, the mesio-buccal cusp of the maxillary first molar occluding anterior to the buccal groove of the mandibular first molar i.e., the maxillary first molar is inline with or anteriorly positioned relative to the mandibular first molar. This most commonly causes a retro gnathic facial profile.

In Class 3, the mesio-buccal cusp of the maxillary first molar occluding posterior to the buccal groove of the mandibular first molar i.e., the maxillary first molar is severely posteriorly positioned relative to the mandibular first molar. This causes a prognathic facial profile.

### 2.13. TMJ Dysfunctions

TMJ dysfunctions are different pathological manifestation related to the temporomandibular joint. Pain, followed by restricted mandibular movement and noises from the joints during jaw movement are the first signs of these conditions.

It is a symptom complex and it is thought to be caused by multiple factors. Occlusion or malocclusion plays a role in the determination of the position of the condyle in the fossa. It has been recognized that pathologic occlusions are associated with four types of abnormal position of the condyle in the fossa. The first type, based on the frequency of occurrence, was distal condylar displacement, associated with anterior interferences of occlusion or by migration of posterior teeth due to missing teeth. The second type of pathosis in the joint was found in those patients in whom the posterior teeth were missing or a clenching habit was present. It was a superior, superior-anterior or supero-posterior overload on the disc and articulating surfaces. This gave rise to the hypotheses of a condition of loss of posterior support.

A third type emerging from the study was that of contralateral interference during chewing motions. These would occur in mesiotrusive movements. Usually these patients showed cross bites.

The last of these types is the position of the condyle too far anteriorly either during the habitual bite or an anterior range of function made necessary by protruding upper incisors. This type occurs usually in class II division 1 patients in which is present a great overjet [2].

There is also an anatomical and genetic predisposition to this pathology, in addition trauma, occlusal changes, parafunction and stress have a determinant role in the onset.

Some conditions are reversible, and related to psychosocial factors like anxiety, depression and anger, instead others are degenerative such as arthritis and arthrosis: These joints are one of the most used in the body so very often wear and degeneration can occur if there is not a good function, and a good occlusion that allow it to protect the integrity of the system.

### 2.14. Spinal Disease

Diseases are not the same and do not have the same importance in reducing patient’s quality of life, some are more recurrent than others, and in general patients refer pain when bone changes put pressure on the spinal cord or nerves; among the most common and less invalidating spinal pathologies, scoliosis is a condition in which the spine acquires a shape of “C” or “S” over three dimensions. In adolescent people, it typically does not cause problems and pain is neither present, but there are severe conditions in which breathing can be affected. Other common spinal pathologies are lumbar spinal stenosis, spina bifida and cauda equine syndrome. Lumbar spinal stenosis is a narrowing of the spinal canal of the vertebrae in the lumbar region, and the result is pain and difficulties to use lower extremities and is common in patients older than 65 years of age; medical management is effective, but laminectomy is a standard in surgical treatments. Spina bifida is a birth defect consisting in an incomplete closure of the spine and membranes around the spinal cord during early development, and in most cases, is incompatible with life. In a patient affected by cauda equine syndrome, the bundle of nerves below the end of the spinal cord is damaged and severe low back pain and saddle anesthesia is present because there is a compression on nerves; surgical management is the solution: The longer is the time before intervention to remove the compression causing nerve damage, the greater the damage caused to the nerve will be.

### 2.15. Posturology

**Figure 1:**
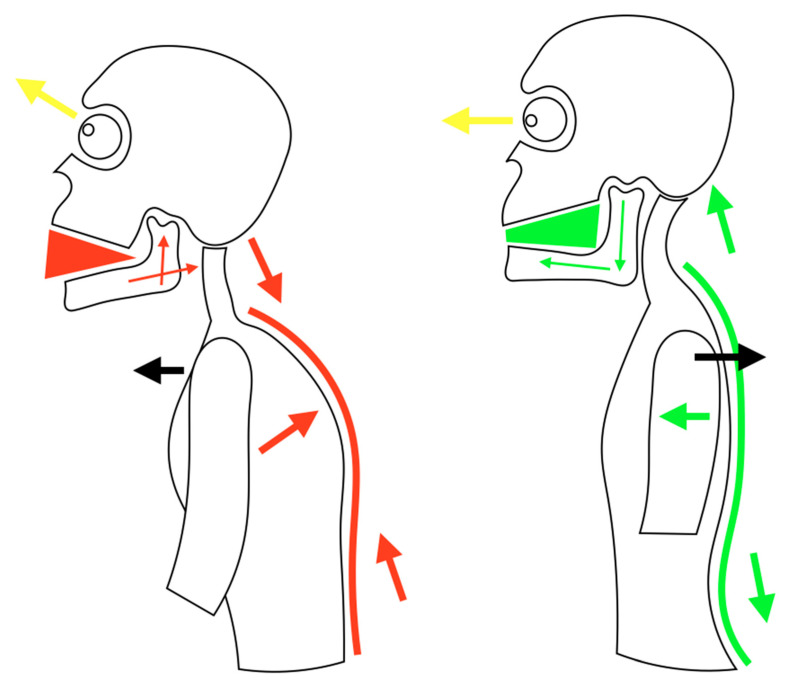
Schematically representation of a condition of postural imbalance on the left and balance on the right. Note how mandible position is strictly related with trunk posture.

Posturology is the scientific study of the human body’s static and dynamic attitude as it balances in space against gravity and other forces. The relationship with the surrounding environment was analyzed and the postural balance is the ability to maintain the body in an upright position against gravity. Posture is organized into two subcomponents based upon the sensory input received from the environment and the functional response of the body to this feedback. The two subcomponents are orientation and stabilization. Orientation is the process of proprioception, due to environmental sensory input. Human body orients in space by recognizing forceful factors such as gravity, and modes of support such as the ground to support the body while upright or a chair to support the body while seated, within the world around us. Stabilization is the process of stiffening one part of the body to allow free movement of another body part within the normal range of motion. An essential scheme of the mechanism just described is shown in Figure 1. If there is an injured part of the body, as in an ankle sprain, the body naturally stabilizes the injured area allowing more movement of other parts of the foot to compensate for the injured area.

## 3. Results

The results obtained from a careful analysis of the literature, after applying the correct inclusion and exclusion criteria, were further evaluated by the authors.

### 3.1. Study Selection

**Figure 2:**
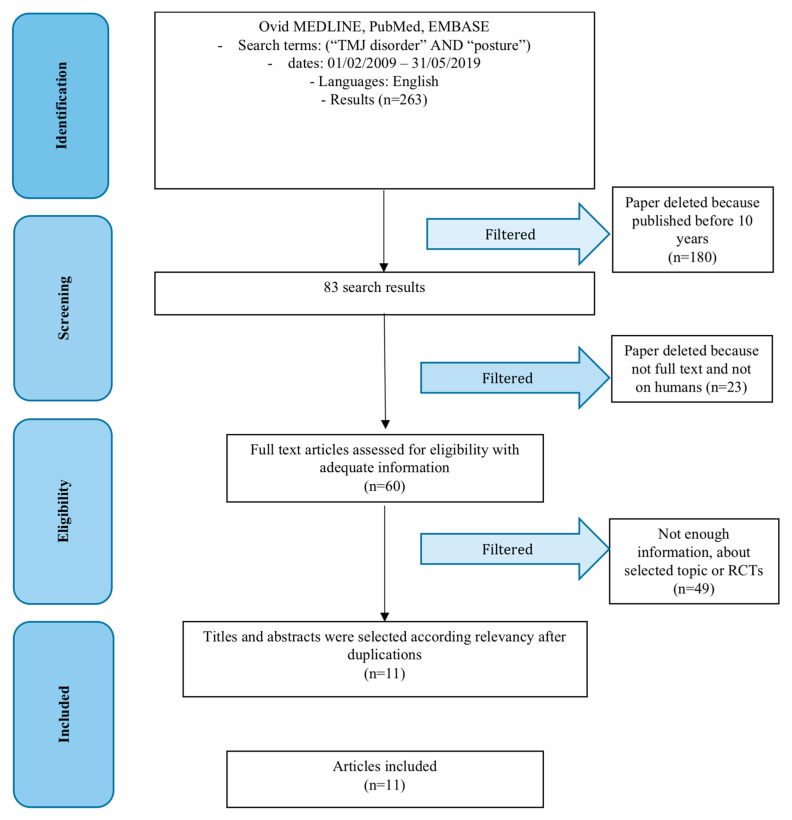
PRISMA (preferred reporting items for systematic reviews and meta-analyses) flow diagram. RCT: Randomized controlled trial.

The initial research led to a high number of articles, 263. Subsequently, applying the exclusion criteria, articles older than 10 years were removed first. At this point the remaining 83 articles were further skimmed by evaluating only the studies on humans (71), and subsequently only those available in full text format (60), so as to be able to consult all the available results. Then we selected manually the articles and at the end of the research 11 articles were obtained, as shown in Figure 2.

### 3.2. Risk of Bias within the Studies

It was not possible to carry out a univocal statistic between the articles as these evaluate different aspects of TMJ disorders, however, the risk of bias was analyzed for each article individually and outlined in Table 1.

### 3.3. Synthesis of Results

Holtz et al. [26] considered different symptoms, such as cervical vertigo, tinnitus, dysphagia and craniomandibular dysfunction and they found that clinically relevant neuroanatomical convergence of the upper cervical spine (occiput to C3) is essential for the interpretation of functional otorhinolaryngological symptoms, because evaluating only our subject of interest, they report that craniomandibular dysfunction is an umbrella term for a functional dysregulation of the masticatory muscles and the temporomandibular joints. Craniovertebral joints and TMJs are strictly related and together form a cybernetic unit regulated by motoric programs via descending corticobulbar and corticospinal tracts. As a result, movements of the temporomandibular joints and the craniovertebral joints are performed in a coordinated fashion. Sperry et al. [27] discovered that, repeated loading and obviously, trauma can induce structural and biochemical changes in joints, and the result is an alteration of the microenvironment and a modification of the biomechanics of their constitutive tissues, which are innervated and could cause severe pain. Their results are complex and well described from a biomechanical and biochemical point of view and they highlight that structural changes to joint tissues and inflammatory cascades, which separately activate pain fibers with effects on thresholds for mechanical injury and/or dysfunction, should be evaluated by clinicians. List et al. [28] showed that temporo mandibular disorder (TMD) affects up to 15% of adults and 7% of adolescents. Chronic pain is the major motivation for patients with TMD to request treatment. TMD can associate with weakened general health, depression and usually disturb the quality of life of the patient. They evaluated the historical background, symptomatology, epidemiology, risk factors, genetics, hormonal factor, pain comorbidity, trauma and parafunctions, occlusal and psychosocial aspects of temporomandibular disorders and clearly described how complex is this pathology. Butts et al. [29] proposed a new three type classification of TMD, Type 1, which comprises muscle disorders, Type 2a/b, which considers disc displacement with and without reduction and Type 3, which includes any joint pain; they also described the key role that the interaction between the lateral pterygoid muscle with both the joint capsule and articular disc, the superior and inferior head had in this pathology and in another article [30] reviewed the current strategies to conservative treatment strategies used to reduce the pain and disability associated with TMD. They reviewed different approaches, such as exercise, soft tissue release, electrophysical modalities, splint therapy, joint mobilization and manipulation, needling modalities and evaluated for each one the evidence supports different therapies. Costa et al. [31] proved that the common neuronal pathways and central sensitization processes are acknowledged as the main factors for the association between TMD and primary headaches; in this work, authors evaluated the relationship between TMD and a headache, cervical region disorders, fibromyalgia and pain mechanisms and they encourage the multidisciplinary and integrated approach, in order to better treat patients and give pain relief to them. Gazit et al. [32] considered TMD in a rare syndrome, the Ehlers-Danlos syndrome–hypermobility type (EDS–HT), and proved that even in these conditions TMJ should be evaluated and treated due to recurrent dislocations and subluxations and chronic pain. They highlighted that many patients affected by this syndrome complain of headaches related to the neck or facial pain, related to jaw or TMD, and these conditions are also be part of dysautonomia, which was found in 78% of EDS–HT patients versus 10% of controls. Findings from Alcantara et al. [33] reveal a theoretical and clinical background based on the discovery of spinal and extraspinal subluxations involving the cervico-cranio-mandibular complex and evaluation of the infant while breastfeeding. They found that chiropractors commonly addressing dysfunctions of the cervico-cranio-mandibular complex with the use of a large number of spinal manipulative techniques including high velocity, low amplitude thrust appropriate for the patient’s age, Gonstead technique, activator methods, Logan basic technique, toggle recoil and the upper cervical technique. They also identified the use of a number of adjunctive therapy techniques including cranial therapy or craniosacral therapy and soft tissue manipulation. Despite this divergence in the clinical approach, their review did find a common theoretical perspective based on the detection and removal of spinal and extra-spinal subluxations that help in treating infants with breastfeeding difficulties by correcting cervico-cranio-mandibular dysfunction. Assouan et al. [34] studied patients with tuberculosis and found that pre-auricular swelling was the predominant functional sign, often without fever or change in the general health status. There were not specific biological and radiological abnormalities. They did not observed lung involvement and the TMJ recovered its normal function after appropriate treatment, that must be administered by a specialized team, and involves especially antibiotics; intra-articular lavage and surgical approaches are not used anymore and currently the only contribution of surgery is diagnostic. Munir et al. [35] used magnetic resonance imaging to evaluate the juvenile idiopathic arthritis and found that this is an accurate diagnostic method for evaluating early and intermediate changes in the TMJ in these patients; with an agreement for the detection of effusion and synovial thickening on unenhanced MRI was 75% and 62.5%, respectively. Davis [36] shows that tissue damage, detected or not by the available diagnostic tools, is probably the main determinant of central hypersensitivity in whiplash injury, that is closely related to TMD: Their results show that neck function is an integral part of natural jaw behavior, and that a neck injury can impair jaw function and therefore disturb eating behavior. This condition stresses a basic importance of linked control of the jaw and neck sensory-motor systems. A neck injury is associated with deranged control of the mandibular and head-neck movements during jaw opening–closing tasks, and therefore might compromise natural jaw function.

## 4. Discussion

During the analysis of the results we were able to highlight some correlations between these pathologies. Furthermore, some articles considered contemplating possible innovative therapeutic techniques for the resolution of these conditions. TMD are complex to treat and every contribution could help clinicians to give the best treatment to their patients. Multidisciplinary approach is the key to success, especially for the different aspects of these conditions that we evaluated in synthesis of the results paragraph. According to Holzl et al. [26] an interdisciplinary cooperation for functional disorders of the upper cervical spine and TMJ is important. Authors for the upper cervical spine mean not a unitary system but a craniovertebral joints (occipit to C3) and lower cervical spine (C3 to C7). The craniovertebral joint region is a sensory organ for the perception of movement and position. Hozl et al. say that bruxism, TMJ pain or TMJ limitation mobility or TMJ sound are correlated to cervical disturbances; TMJs and craniovertebral joints form a cybernetic unit that is regulated by neuromuscular and neurophysiological reflexes. Myofunctional disturbances of the TMJs and craniovertebral joints may lead to overlapping complaints. Treatments consist of myofunctional dental treatment and manual therapy. According to Sperry et al. [27] joint micromovement, and peripheral pain sensors activated, can contribute to chronic pain with central nervous system alterations, and jaw pain typically initiated by repeated atypical loading of the temporomandibular joint (TMJ) that leads to inflammatory cascades within the synovium and that can sensitize pain fibers in the joint. According to List et al. [28] etiology of TMD is complex and not clearly understood. TMD can be improved through noninvasive therapy, behavior therapy, pharmacotherapy, physical therapy and occlusal appliances. In their review they highlighted that a staged approach is more effective in treatment and the importance of reversible treatment. In a study of Butts et al. [29] temporomandibular dysfunction is a multifactorial condition that affects TMJ and masticatory muscles, resulting in pain and disability in 5%–12% of the population. They proposed a three type classification of TMD: The first type related to muscle, the second type related to disc displacement and the third type related to joint pain. Authors well reviewed literature, searching about etiology, and muscular activity and their new classification confirms precedent findings about TMD onset, in particular it stresses the key role that the superior and inferior head of the lateral pterygoid muscle have. In another study, the second part of Butts et al. [30], TMJ disorders pain or disability can be reduced through dry needling or acupuncture of the lateral pterygoid and posterior. There was limited evidence for manual soft tissue work of muscle of mastication, which may be hard to access, the same evidence is for laser therapy, ultrasound, iontophoresis or TENs. According to the authors the goal is to impact anatomic structures directly related to etiology of TMD, in order to carry on conservative and reversible treatments that allow clinicians to relieve pain of patients. Costa et al. [31] evaluated the association between TMDs and headaches or cervical spine dysfunction. The biomechanical aspects are not the only cause of relationship through TMD and cervical spine dysfunction, neuronal convergence of the trigeminal and cervical sensory pathways need to be mentioned too; in fact, TMD involves more than a single clinical entity and rather encompasses a group of musculoskeletal conditions affecting the masticatory system, expresses its complexity through the concept of comorbidity. From their results, migraine and chronic fatigue syndrome increases TMJ pain and worsens the symptoms of TMD, but even psychological conditions, such as anxiety and depression have an important role on this issue and increase the patient's perception of pain and muscle fatigue. Gazit et al. [32] showed that the Ehlers-Danlos syndrome carries a high potential for disability due to frequent dislocation and subluxations and chronic pain of joints. Authors found that even in a complex syndrome, the perception of discomfort and the loss of autonomy related to TMD is considerable, almost eight times more respect controls. Postural instability, balance and gait impairment, resulting in increased frequency of falls is a problem for these patients and authors wrote that impaired proprioceptive acuity influences muscle strength; they suggest that improving muscle strength on the basis of proprioceptive impairment may be more important for reducing activity limitations than just improving muscle strength. Alcantara et al [33] evaluated care of infants with breastfeeding difficulties by addressing spinal and extraspinal subluxations in the cervico-cranio-mandibular complex. This review evaluated correlation between TMD, cervico dysfunction and breastfeeding on infants. Major findings from authors are related to different techniques from chiropractors, and the final results is that detecting and removing spinal and extra-spinal subluxations on the treatment of infants with breastfeeding difficulties, correcting cervico-cranio-mandibular dysfunction is more effective. According to Assouan at al. [34] even in rare pathology, like extra-pulmonary and extra-spinal tuberculosis, TMJ involvement could be present and it must be considered in the differential diagnosis for TMJ disease. This occurrence may seem rare or inconsistent, but globalization and the increase of migratory flows have led to hospitals pathologies that appeared to have disappeared. Authors wrote that today the gold standard of treatment is antibiotics and the only contribution of surgery is diagnostic, or useful to treat complications or sequels. Munir et al. [35] used magnetic resonance imaging to evaluate the juvenile idiopathic arthritis and found that this is an accurate diagnostic method for detecting this pathology. Their valuable contribution is useful to bring order to the classification of joint pathologies visualized with MRI, the best current diagnostic tool for the diagnosis of the TMD. Davis [36] evaluated the mechanisms of chronic pain and found that spinal cord hyperexcitability in patients with chronic pain after a whiplash injury can cause exaggerated pain following low intensity nociceptive or innocuous peripheral stimulation. A neck injury is associated with deranged control of the mandibular and head-neck movements during jaw opening–closing tasks, and therefore might compromise natural jaw function. A clinical implication is that examination of jaw function should be recommended as part of the assessment and rehabilitation of whiplash patients.

The stability of occlusion depends on the position of the teeth, the premolars and their anatomical position, are essential for having efficient mastication as they support the occlusal balance and are central in keeping the vertical dimension of the face morphology. [37]. An excessive muscular tension determines an anomalous mechanical load on the joint that causes pain and discomfort to patients, and even on the teeth and on the fillings or prosthesis eventually existing in the oral cavity of the patient, exposing dental elements to a higher risk of fracture.

## 5. Conclusions

It is evident that there is a correlation between TMJ and posture, therefore an overall evaluation, carried out by an orthodontist and an orthopedist is necessary to better evaluate the patients’ needs and establish treatment priorities especially in symptomatic and in growing patients. A multidisciplinary approach is the key to success because TMJ has a central role in the cybernetic unit of the head and neck; no passage, from clinical diagnosis to radiological examination, from conservative treatment to surgical treatment can be underestimated and even treating the psychologically aspect is relevant for the benefit of every patient.

## Figures and Tables

**Table 1:** Risk of bias results evaluation.

Author (Year)	Risk of Bias			
	Unclear	Low	Moderate	High
Holtz et al. (2019) [26]		X		
Sperry et al. (2017) [27]		X		
List et al. (2017) [28]			X	
Butts et al. (2017) [29]			X	
Butts et al. (2017) [30]			X	
Costa et al. (2016) [31]		X		
Gazit et al. (2016) [32]			X	
Alcantara et al. (2015) [33]		X		
Assouan et al. (2014) [34]			X	
Munir et al. (2014) [35]			X	
Davis et al. (2013) [36]		X		
